# Supplementary material for: Analgesic effectiveness of wound infiltration with bupivacaine versus a mixture of bupivacaine and tramadol for postoperative pain management among parturients undergoing elective cesarean section under spinal anesthesia: A randomized controlled trial
Source: PLoS One. 2025 Nov 12;20(11):e0336372. doi: 10.1371/journal.pone.0336372 (PMC12611141; doi:10.1371/journal.pone.0336372)
Supplement: S1 Text — (PDF) [file pone.0336372.s003.pdf]

Study protocol in English

Pain severity measurement

Numeric Rating scale

The scale will be taken 5 times within the first 24 hours. Patients will be asked to rate their pain will be assessed and recorded at different points in time. The patient will be asked to say what number on a 0 to 10 scale would you give your pain right now. If the explanation suggested above is not sufficient for the patient, further explanation or conceptualization of the scale will be done:

0 = No Pain

1-3 = Mild Pain

4–6 = Moderate Pain

7-10 = Severe Pain

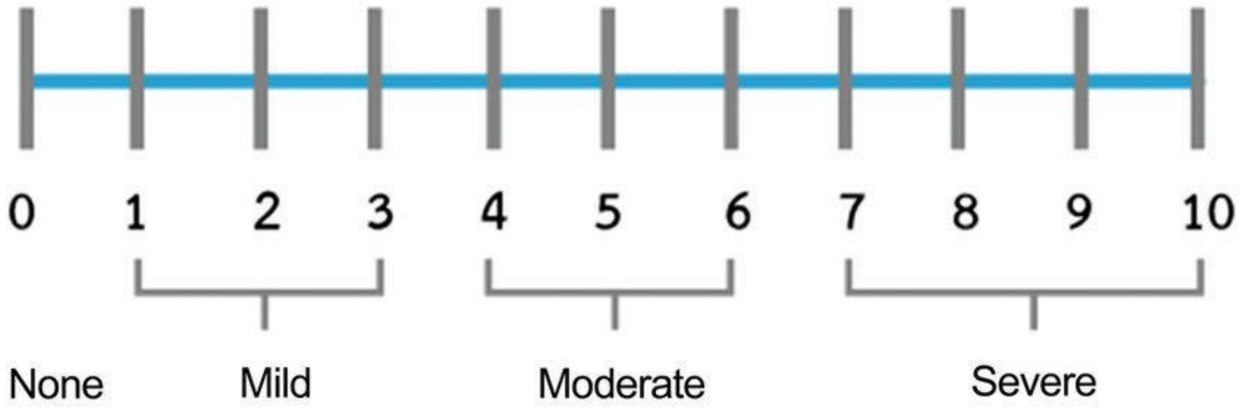

Figure 1. Numeric Rating Scale (NRS)

A. The scale will be taken 5 times within the first 24 hours. The patient will be asked one of the following questions:

    a) What number on a 0 to 10 scale would you give your pain right now?

B. When the explanation suggested above is not sufficient for the patient, further explanation or conceptualization of the scale will be done:

The patient pain management protocol

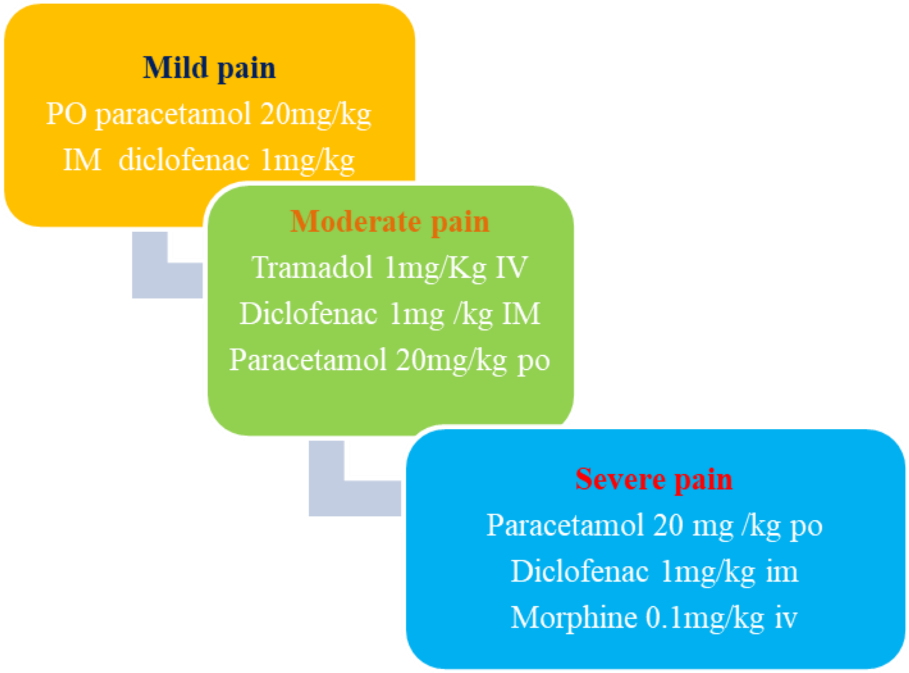

2. **The severity of postoperative nausea, vomiting**

Incidence of PONV, which was evaluated by asking the patients to grade their nausea and vomiting according to a 3-point scale:

0=no nausea, vomiting

1=nausea only

2=retching and/or vomiting.

3. **Management protocol for intraoperative nausea and vomiting**

Metoclopramide 10 mg IV with dexamethasone 6-10mg IV

4. **Failed spinal anesthesia management protocol**

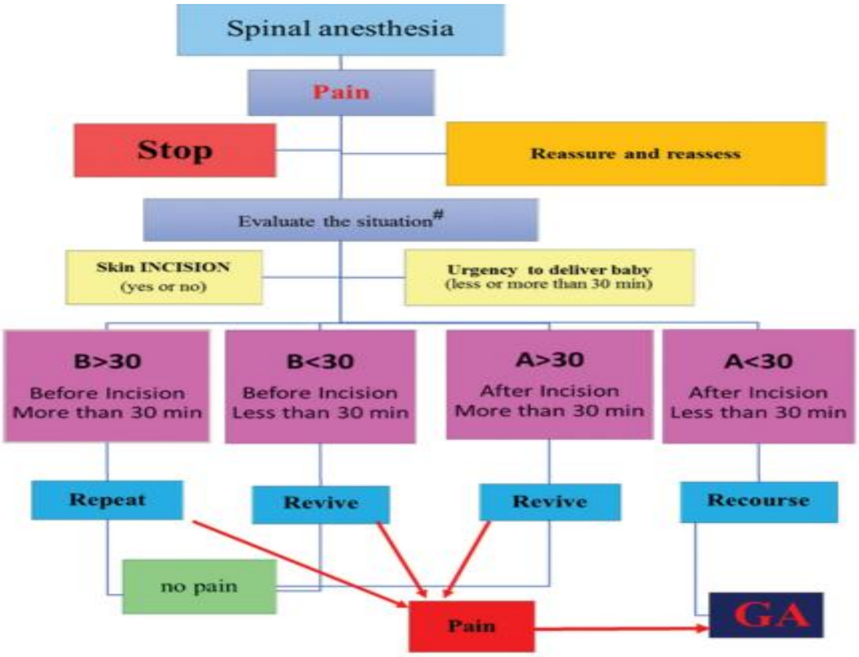

Figure 2: Algorithm: a safe approach to failed spinal anesthesia(29).

Assess Difficult Airway, Starvation Status, Technical Difficulty in performing SA, and Comorbidity. GA=General anesthesia

Shivering grading and Management

Grade	Clinical sign
0	No shivering
1	One or more pilo erection, peripheral cyanosis without other cause
2	Visible tremor involving only one muscle group
3	Visible tremor involving more than one muscle group
4	Gross muscular activity involving the whole body

=>Tramadol 0.5mg/kg for patients having a shivering grade greater or equal to 3.

PONV prophylaxis and scoring the (APFEL score)

Nausea vomiting degree	Score
No any complaint	0
Mild degree nausea	1
Moderate degree nausea and vomit	2
Frequently vomit	3
Severely (continuously) vomit	4

PONV prophylaxis based on APFEL score			
Risk score	Prevalence of PONV	Prophylaxis No of anti-emetic	Drugs for treatment
0	9%	0-1	Ondansetron 4mg
1	20%	1	Ondansetron 4mg<br>Dexamethasone 4mg
2	39%	2	Ondansetron 4mg<br>Dexamethasone 4mg<br>Propofol infusion
3	60%	3	Ondansetron 4mg<br>Dexamethasone 4mg<br>Propofol infusion<br>Scopolamine patch
4	78%	4	Ondansetron 4mg<br>Dexamethasone 4mg<br>Propofol infusion<br>Scopolamine patch

PONV prophylaxis APFEL score with management at Dilla University General Hospital

Hypotension management protocol

Events	Intervention guides	Remarks
A drop in blood pressure less than 20% from baseline or a drop in SBP but, >80mmHg	Rapid fluid infusion and administration of oxygen by facemask at 3l/min	
A drop in blood pressure more than 20% of baseline or SBP <80mmHg.	Give 5 mg of ephedrine together with fluid infusion	Repeat if not responding
If not responding to ephedrine after a maximum dose of 20mg.	Give epinephrine<br><br>1ug/kg as bolus together with fluid infusion	Repeat every 3-5 minutes

Data accuracy check sheet

Code of group allocated __________________patient ID_____________

S. NO.	Tools	Yes	No
1	Are all questions on Socioeconomic and demographic data filed appropriately?		
2	Are all questions on the preoperative period filled appropriately?		
3	Are all questions on the intraoperative period filled appropriately?		
4	Are all questions on the postoperative period data filled appropriately?		

Name of the data collector……………………………… Signature…………….

Name of supervisor ………………………………… Signature……………

Date ……………………………..

Patient safety check sheet

Code of group allocated ____________________Patient ID _________________

**NO**	**Tools checked**	**Yes**	**No**	**Data entry**
**1**	Are the Inclusion criteria /exclusion criteria Appropriately done?			

**2**	An informed consent issues sheet was given and the patient understood.			
**3**	Is the consent sheet signed?			
**4**	Was randomization done appropriately?			
**5**	Are all rescuing vasopressors and antiemetic's prepared			
**6**	Did the data collectors follow the steps correctly?			
**7**	Were any complications assessed and managed?			
