# Supplementary material for: Analgesic effectiveness of wound infiltration with bupivacaine versus a mixture of bupivacaine and tramadol for postoperative pain management among parturients undergoing elective cesarean section under spinal anesthesia: A randomized controlled trial
Source: PLoS One. 2025 Nov 12;20(11):e0336372. doi: 10.1371/journal.pone.0336372 (PMC12611141; doi:10.1371/journal.pone.0336372)
Supplement: S1 Table — (DOCX) [file pone.0336372.s004.docx]

**Appendix: Pain severity at different time intervals between two groups**

|  | | **Follow up of pain by NRS at 2hr** | | | |
| --- | --- | --- | --- | --- | --- |
|  | | None | Mild | Moderate | Severe |
| Intervention given | B(n=30) | 5 | 25 | 0 | 0 |
|  | BT(n=30) | 28 | 2 | 0 | 0 |
|  | **Follow up of pain by NRS at 6hr** | | | | |
|  |  | None | Mild | Moderate | Severe |
|  | B(n=30) | 1 | 29 | 0 | 0 |
|  | BT(n=30) | 12 | 18 | 0 | 0 |
|  | **Follow up of pain by NRS at 12hr** | | | | |
|  |  | None | Mild | Moderate | Severe |
|  | B(n=30) | 0 | 30 | 0 | 0 |
|  | BT(n=30) | 6 | 24 | 0 | 0 |
|  | **Follow up of pain by NRS at 18hr** | | | | |
|  |  | None | Mild | Moderate | Severe |
|  | B(n=30) | 0 | 0 | 1 | 29 |
|  | BT(n=30) | 13 | 16 | 0 | 1 |
|  | **Follow up of pain by NRS at 18hr** | | | | |
|  |  | None | Mild | Moderate | Severe |
|  | B(n=30) | 0 | 30 | 0 | 0 |
|  | BT(n=30) | 1 | 29 | 0 | 0 |

Where, B= bupivacaine alone group; BT=bupivacaine and tramadol group; n=number of participants
