# Supplementary material for: Analgesic effectiveness of wound infiltration with bupivacaine versus a mixture of bupivacaine and tramadol for postoperative pain management among parturients undergoing elective cesarean section under spinal anesthesia: A randomized controlled trial
Source: PLoS One. 2025 Nov 12;20(11):e0336372. doi: 10.1371/journal.pone.0336372 (PMC12611141; doi:10.1371/journal.pone.0336372)
Supplement: Attachment — Submitted filename: Response Letter to Reviewers.docx [file pone.0336372.s006.docx]

**Rebuttal Letter**

**Dear Editor,**

We would like to thank the editor and reviewers for their careful and thorough reading of this manuscript and for their thoughtful comments and constructive suggestions, which helped us to improve the quality of this manuscript. We have read the research entitled “Analgesic Effectiveness of Wound Infiltration with Bupivacaine versus a Mixture of Bupivacaine and Tramadol for Postoperative Pain Management among Parturients Undergoing Elective Cesarean Section under Spinal Anesthesia”: A Double-Blind Randomized Controlled Trial

We have made all necessary corrections to the comments given by the editor and reviewers one by one as mentioned below. In addition, spelling, grammar, and syntax errors have been corrected.

**Editors comments**

**Comment 1:** Please adhere to the guidelines for reporting randomized controlled trials; see author's instructions.

**Response:** Thanks for the comment. We have followed the author's guidelines to adhere to the guidelines for reporting randomized controlled trials and corrected it.

**Comment 2:** Report details on how informed consent for the research was obtained (or explain why consent was not obtained).

**Response:** Thanks for the comment.  We have described how the consent was taken in detail as “The parturients were informed about the study's purpose, procedures, and potential risks and benefits. Informed written consent was obtained from the selected parturients to confirm their willingness to participate in the study. To maintain confidentiality, the parturients' names were not included in the written questionnaire. The parturients were assured that their refusal to consent or withdrawal from the study would not affect or jeopardize their access to care.”

**Comment 3:** NRS and VAS are reported. These outcome measures are not interchangeable. Please explain which was used to measure pain intensity and how this affected the outcome.

**Response:** Thanks for the comment. It was an editorial problem; we have corrected it. We have assessed pain severity with the numeric rating scale.

**Comment 4:** Follow the author's instructions on how to submit figures and tables.

**Response:** Thanks for the comment. We have followed the author's instruction and corrected it.

**Reviewer #1**

**Comment 1:** The power calculation section needs some clarity. Clarify the analgesic consumption in the two groups (what unit of measurement) and the power level and alpha at the start, and then report the numbers needed to pick up the hypothesized difference (is it 60?). then add the 10% for loss to follow up. I dont' follow how you start with 30+30, then add 10% and end up with 60.

**Response:** Thanks for the comment, We have used G-power to calculate the sample size. The sample size was determined by selecting analgesic consumption (N1 = 30, µ1 = 386.17mg, SD1 = 233.84mg for a group consisting of bupivacaine and tramadol, and N2 = 30, µ2 = 192.50mg, SD2 = 134.77mg for bupivacaine alone). By utilizing alpha 0.05, effect size 0.4525, power 90% (1-β), and sample size determined using power analysis with G power software (version 3.1.9.7) to be 60. We added 10% for the possible dropout rate to the final sample size, which was 53. Then the sample size was increased to 60 in total, and thirty parturients were allocated to each group. Thank you in advance, We have corrected the analgesia consumption between two groups. The mean analgesic consumption of drugs in milligrams (mg) consumed in the first twenty-four hours following the surgery between these two groups to manage pain after assessment.

**Comment 2:** The repeated measures approach is appropriate, but the section was a bit confusing. It seems like a single repeated measures mixed linear regression model was used? Clarify the specifics of the model, e.g., clustered errors.

**Response:** Thank you for your comment. We totally agree with you. It was confusing because during the study we considered both VAS as a continuous variable and analyzed it with mixed linear equations and NRS as a categorical variable and analyzed it with general estimating equations to assess whether these two have any difference; however, there was no significant difference between these two pain assessment tools, so we have only used NRS (categorical) for pain assessment and analyzed it with general estimating equations. Now we have addressed all those issues.

**Comment 3:** There is no information on missing data. Was the data complete for all patients in both groups? Please state so. If not, would multiple imputation approaches be relevant here?

**Response:** Thank you for comments. We have corrected it; it was an editorial problem. There was no missing data, and the data was complete for both groups; we have mentioned it in the result section (page 7) and figure 1.

**Reviewer #2**

**Comment 1:** The authors aim to compare the effect of wound infiltration (WI) with tramadol and bupivacaine versus bupivacaine on its own on pain in women after CSection. They write that previous studies assessed wound infiltration with bupivacaine and tramadol with conflicting results. They cite: (1) Sarwar A TS. Effectiveness of Local Bupivacaine Wound Infiltration in Post Operative Pain Relief After Caesarean Section. J Soc Obstet Gynaecol Pak. 2016;6(3):125-8 and (2) Haliloglu et al . Analgesic efficacy of wound infiltration with tramadol after cesarean delivery under general anesthesia: Randomized trial. J Obstet Gynaecol Res. 2016 Jul;42(7):816-21. I reviewed the abstracts of both papers VERY briefly – they both seem to have found that WI with tramadol effective. I am, therefore, not sure where the results are in conflict with each other. The authors might expand on this point. This study seems to be a replication of the study carried out by ref 18 : Sachidanandaet at al. Comparison of Analgesic Efficacy of Wound Infiltration with Bupivacaine Versus Mixture of Bupivacaine and Tramadol for Postoperative Pain Relief in Caesarean Section Under Spinal Anaesthesia: A Double-Blind Randomized Trial. Journal of Obstetric Anaesthesia and Critical Care 7(2):p 85-89, Jul–Dec 2017. It’s a replication in terms of the dose of tramadol that was infiltrated into the wound; size of cohort, method of anesthesia (spinal). Replicating another study is certainly ethical, but this should be clearly indicated when describing the aims of the study in the introduction.

**Response:** Thank you for comments, various studies have been conducted in this area, but specifically on cesarean section there are a few studies conducted and no studies was conducted in our country; furthermore those conducted studies have used different analgesics in addition to their intervention, different pain assessment tools, and older analysis method. To determine the effectiveness of intervention, we have calculated the effect size which shows clinical significance, in which none of the previous studies have calculated. We have used Generalized estimating equation (GEE) which has several advantages including flexibility in correlation structure, handling of missing data robustly, focus on population-Averaged effects, and computational effeciecy. None of previously conducted used this analaysis model. Thank you in advance for your observation, we have added the detailed information in introduction section.

**Comment 2:** Third paragraph – I suggest that caring for pain after C-Section is complex for the reasons that the authors list but probably a major factor is that mothers, wishing to breastfeed their newborns, and medical and nursing staff are not well acquainted with the medications which are safe vs less safe for lactation. I suggest that the authors refer to the review by Patricia Lavand’homme Lavand'homme P. Postoperative cesarean pain: real but is it preventable? Curr Opin Anaesthesiol. 2018 Jun;31(3):262-267. The authors could use information from this review to describe the complexity of caring for women post CSection, including addressing poor recovery and long long outcomes.

**Response:** Thank you for your suggestion, We have added it, as “Postoperative pain remains a principal concern for parturients undergoing cesarean sections. A cause of suffering, as it can compromise timely mother-baby bonding and delay recovery, despite the advancements of Enhanced Recovery After Surgery (ERAS) programs. The risk of depression and chronic postpartum pain was independently correlated with the severity of acute postpartum pain, but not with the mode of delivery. Compared to women who experienced mild postpartum pain, those who experienced severe acute pain were two times more likely to experience persistent pain and three times more likely to develop postpartum depression”

**Comment 3:** According to Gesseck et al Neonatal Exposure to Tramadol through Mother's

Breast Milk. JAnal Toxicol. 2021 Sep 17;45(8):840-846. , use of tramadol during pregnancy is

generally avoided and may cause some reversible withdrawal effects in neonates, and its use

during lactation is not licensed by the manufacturer. I assume that if the tramadol is infiltrated to

the incision, absorption to the blood stream will be less compared to when the medication is

administered systemically. Later in the manuscript, the women seem to have received oral

tramadol – and so this might not comply with the current recommendations for lactating women.

The authors should address the issue of which opioids are recommended vs not for women after

C-Section and that there are warnings regarding use of tramadol. I am aware that different

guidelines, internationally, recommend different analgesics for lactating mothers. I tried to look

up international guidelines for recommendations as to which analgesic medications are safe for

lactating women. I cite only a few examples here – as to this this thoroughly would require much

more work , from the little I have seen, there is little standardization among the guidelines.

LactMed® database – recommend using tramadol for the shortest time possible. WHO

guidelines for lactating mothers BREASTFEEDING AND MATERNAL MEDICATION

Recommendations for Drugs in the Eleventh WHO Model List of Essential Drug – the most

recent version seems to be from 2002. They do not mention tramadol among the opioids

Roofthooft et al PROSPECT Working Group of the European Society of Regional Anaesthesia

and Pain Therapy. PROSPECT guideline for elective caesarean section: an update. Anaesthesia.

2023 Sep;78(9):1170-1171.– recommend wound infiltration but with a local anesthetic alone.

The authors write that tramadol has local anesthetic properties when it is injected into peripheral

nerves, citing REFS 13 and 14. I looked up these references. Reference 14 - Subedi et al . An

overview of tramadol and its usage in pain management and future perspective. Biomed

Pharmacother. 2019 Mar;111:443-451 – states in the Introduction … that use of tramadol is …

restricted in pregnant women as well as breast feeding mothers as it may cause birth defects and

harm the foetus and to the nursing babies. Tramadol is rated as Category C in the pregnancy risk

drug by American Food and Drug Administration. Due to these effects, physicians hould avoid

prescribing tramadol to pregnant women and nursing mothers. This citation does certainly not

advocate using tramadol in lactating mothers. Citing it for this statement was probably

unintentional. The second reference seems correct for this statement: Kaki AM, Al Marakbi W.

Post-herniorrhapy infiltration of tramadol versus bupivacaine for postoperative pain relief: a

randomized study. Annals of Saudi medicine. 2008;28(3):165-8. 14.

**Response:** Thank you for your comment; We totally agree with your concern, it was also our

concern. Here we have administred tramadol subcutaneously which have very less effect on

mothers due its minimal absorption to the blood stream. According to literatures, if the tramadol

is infiltrated to the incision site subcutaneously, absorption to the blood stream will be less

compared to when the medication is administered systemically.All patients allocated to

intervention group(Bupivacaine and Tramadol) received 0.7 mg/kg of 0.25% bupivacaine and 2

mg/kg of tramadol. Neither control nor intervention group received oral tramadol. Thanks in

advance we have corrected reference 14, it was unintentional.

**Comment 3:** Why did the authors use both VAS and NRS for assessing pain? Was pain assessed

at rest or movement-related? Which medication was injected into the spinal catheter? What do

the authors intend to say when they write? At the 6th hour postoperatively, the time the uterus

stayed outside the abdomen and pain severity was statistically significant (8.22±1.767),(P=0.027)

**Response:** Thank you for your comment; We have corrected it, only NRS was used to assess

postoperative pain. The postoperative pain was assessed during rest time.

Spinal anesthesia was performed in a sitting position with 0.5% hyperbaric bupivacaine with a

dose of 0.07 mg/height.

We have assessed for the association between uterine exteriorization and pain severity and there

are evidences that reveals uterine exteriorization have effect on post-operative pain severity. The

studies postulates that, this pain is primarily visceral pain, mediated by C-fibers that transmit

pain signals from the uterus to the brain. Uterine exteriorization also affects the vagus nerve,

which innervates the uterine wall, potentially inducing nausea and vomiting. In our study there

was statistically significant association(8.22±1.767), (P=0.027) between post-operative pain

and uterine exteriorization at 6^th^ hour between two groups.

**Comment 4:** The authors provide summarized results of the pain readings comparing the two

groups. They should include a table in which the raw pain scores for each of the assessment time

points, for both groups. Or a graph of the pain scores as a function of time. Rather than calculate

means - which can be misleading - the authors could evaluate the proportion of women who

reported scores of eg 3/10 and less at the different time points. I cannot find figure 2.

It will be easier to assess the outcomes related to pain - once the authors address the issues

above. Under Analgesic consumption , page 10 - it seems that patients received systemic

tramadol in addition to the infiltration – is this correct? If so - then this seems to contradict

recommendations and then it is dubious whether publishing the findings from this study is ethical

without extensive discussion of the pros and cons of using tramadol in this population.

**Response:** Thank you for you comment, we have used generalized estimating equations (GEE),

which is advanced analysis model,that mainly focus on population-Averaged effects, rather than

considering single point pain score. This is one of GEEs major advantage over ANOVA.

We have provided figures which are color coded for easy identification from the analysis model,

described details at footnotes, and also made the raw data available as supplementary

information, so the readers can easily access it. The patient have received analgesics in

postoperative period, after appropriate assessment of pain and each given analgesics was

documented. It was not in addition to wound infiltration but if they(bupivacaine alone or a

mixture of bupivacaine and tramadol group) feel pain during postoperative period,

analgesics(rescue analgesics) are given, based on the severity of pain based on patient

management protocol,and finally it was documented and analyzed as total analgesic consumption

in the first 24 hours.

**Comment 5:** Page 14-the authors write Tramadol may prove predominantly useful in patients

with limited cardiopulmonary function, including the aged, the obese, and smokers, in patients

with impaired liver or renal function, and in patients in whom non-steroidal anti-inflammatory

drugs are not suggested or need to be used with caution (26). However these patient groups are

not included in the current study which focuses on C-Section and according to several sources

tramadol – at least systemic – is not recommended for women who are breast feeding. As I wrote

above - this is an issue that should be discussed at some length if this manuscript is to be

accepted for publication.

**Response:** Thank you for your comment, We totally agree with your concerns, here we have

administred tramadol subcutaneously which have minimal effect on mothers due its least

absorption to the blood stream. According to the literatures, if the tramadol is infiltrated to the

incision site, absorption to the blood stream will be less compared to when the medication is

administered systemically. Administration of larger doses opioid is associated with various

systemic effects like nausea and vomiting, respiratory depression, risk of abuse, and economic

crises especially in resource constrained set-ups. These side effects may be doubled or tripled in

patients having respiratory and cardiac comorbidities. We have only discussed to show the

importance of addition of this opioid analgesics in our study tramadol to local anesthetics, but we

have not analyzed this in our study, so we have mentioned it as limitation of our study and

recommended the researchers to conduct well-designed study in this area.

**Reviewer #3**

**Comment 1:**  In the introduction section, I recommend including the prevalence of caesarean

sections in Ethiopia, as the study was conducted there and highlights the burden of pain

management in caesarean sections and the limited access to opioid analgesics and epidural

analgesia in low-resource settings.

**Response:** Thank you for comments, we have added it, as “The prevalence of CS in Ethiopia

was 21% in Butajira General Hospital in 2019; 29.7% in Jugal Hospital in 2019; 47.6% in Dessie

town hospitals; 34.3% in Hospitals in Harar; 30.9% public hospitals in Northern Ethiopia; 24.7%

in Durame General Hospital; 28.1% in three Hospitals of Southwest Ethiopia ; 27.6% in Atta

hospital; 38.5% in a national cross-sectional study; 3.5% in another community-based cross-

sectional study.”

**Comment 2:**  Similarly, the authors include, “Research has revealed that tramadol has certain

local anesthetic properties when it is injected into peripheral nerves (13, 14).” However, the fact

is that wound infiltration, or local infiltration analgesia, involves injecting local anesthetic

directly into the tissues surrounding the wound, not the nerves themselves. Therefore, please

include references that show the effect of tramadol on wound infiltration if possible. Otherwise,

paraphrase the statement if there is a lack of evidence in this regard.

**Response:** Thank you for comments, we have corrected the statement as “Research has revealed

that tramadol has certain local anesthetic properties when injected into peripheral nerves;

however,further studies are needed to reach a definitive conclusion.”

**Comment 3:**  Use a consistent term throughout the documents, either "pregnant" or "parturient."

Additionally, use the same abbreviation to describe the two groups (bupivacaine versus a

comparison of bupivacaine and tramadol) consistently throughout the documents.

**Response:** Thank you for comments, we have corrected it.

**Comment 4:**  Authors mentioned that they used a visual analogue scale and a numeric rating

scale to measure pain severity. What is the importance of using two pain assessment scales for a

homogenous population?

**Response:** Thank you for comments, initially we have planned to assess pain everity with both

NRS and VAS, however there was no significant difference between two groups. Now we have

corrected it and only NRS was used.

**Comment 5:**  Redefine total analgesic consumption in the operational definitions as you are

using mean tramadol consumption quantity instead of saying analgesic drugs in milligrams.

**Response:** Thank you for comments, we have redefined it. We have re-defined total analgesic

consumption as the mean analgesic consumption of drugs in milligrams (mg) consumed in the

first twenty-four hours following the surgery.

**Comment 6:**  You mentioned that the anaesthetist in charge, who was not part of the study,

prepared the medications. Would you write the details of the person who performed the

infiltration procedures? Is it the same obstetrician? Include details of the anatomical locations

and techniques where infiltration was performed.

**Response:** Thank you for comments, We have described it in detail. Following the surgery, the

last vital sign was documented, and the wound site was at lower abdomen and which was

transverse, and about 1-2 inch above the pubic hairline was infiltrated with 0.7 mg/kg of 0.25%

bupivacaine diluted to 20 ml for group B. The BT group received a mixture of 0.7 mg/kg of

0.25% bupivacaine and 2 mg/kg of tramadol diluted to 20 milliliters by the four experienced

obstetricians. The patients and data collectors were blinded, and four equally qualified senior

anesthetist’s in charge, who was not part of the study, prepared the medications.

**Comment 7:**  The authors mentioned that patients received IV tramadol at a dose of 0.5 mg/kg if

they had a shivering grade of three or above. Does this affect your outcome variables?

Intraoperative tramadol may prolong the duration of postoperative analgesia and reduce the mean

consumption of postoperative analgesics. At the very least, please acknowledge this point in the

limitations section. This might be the reason for the discrepancy with other studies that you have

been mentioning in the discussion sections, especially with Sachidananda et al. on the severity of

pain scores at different points in time.

**Response:** Thank you for your observations, Yes it may affect our outcome and we have

mentioned it in the limitation section.

**Comment 7:**  Under the Anesthetic Management and Surgery section, you mentioned that a

numeric rating scale was used to assess the severity of postoperative pain for 24 hours at T2, T6,

T12, T18, and T24 hours, where T represents time. However, in the abstract section, you refer to

both a visual analogue scale and a numeric rating scale to measure pain severity, and you also

operationalize the definitions of both pain assessment tools. Please clarify which assessment tool

you used in the manuscript; probably, you have used NRS.

**Response:** Thank you for comments, we have corrected it. Numeric rating scale (NRS) was used

to assess pain severity for 24 hours which was at T2, T6, T12, T18, and T24 hours-where T is the

time.

**Comment 8:**  Please clarify in the outcome section when you evaluated the incidence of

shivering during the postoperative period, as the current statement does not specify whether it

refers to the intraoperative or postoperative phase. In the results section, the previous caesarean

section scar was an indication for caesarean delivery. Do you think having a CS scar affects your

study outcomes, and do you consider it a limitation?

**Response:** Thank you for comments, we have corrected it as, “A 5-point rating system was used to assess intraoperative nausea and vomiting, and protocol-compliant care was given to any complications.” Thanks in advance for your deep observation, previous cesarean scar has an impact on pain severity due to neovascularization especially when there is repeated cesarean section scar. We have added it to the limitation section.

**Comment 9:**  In the intraoperative characteristics, you mentioned that “At the 6th hour postoperatively, the time the uterus stayed outside the abdomen and pain severity was statistically significant (8.22±1.767), (P=0.027).” This information should not be included here. Please either change the heading to "perioperative characteristics" or move this information to another section.

**Response:** Thank you for comments, we have corrected it.

**Comment 10:** In the discussion section, the authors mentioned that “Similar to the findings of

Sachidananda et al., our study showed a statistically significant difference (p = 0.0484) between

the mean (±SD) postoperative pain score of the treatment group at 6 and 12 hours after surgery

(2.60 ± 0.97) and that of the control group. However, the mean (±SD) postoperative pain score at

18 and 24 hours was 2.07 ± 0.64 and 2.07 ± 0.37 in the groups receiving bupivacaine and those

receiving a combination of bupivacaine and tramadol, respectively, with P= 0.8825(18). The

possible explanation for these discrepancies may be the differences in the study population.”

However, there is no discrepancy in the study populations; both are pregnant women who

underwent caesarean delivery and received similar pain management. The only difference is the

study location. Please provide other plausible evidence if there is a difference in preoperative

analgesics used, doses, and postoperative pain management practices. Again, the authors noted

that “According to another study by Gebremedhin et al., which involved 120 patients undergoing

lower abdominal surgery, the median (IQR) pain severity (NRS) score was 0.0 (0-0) until 12

hours, and 1 (0–5) and 3 (1–5) at 18 and 24 hours in the local wound infiltration (LWI) groups

with bupivacaine and tramadol (BT) compared to LWI bupivacaine alone (B) in the post-

anesthesia care unit and ward. There was a notable difference in pain severity between the BT

and B groups at the twelve-, eighteen-, and twenty-four-hour marks (p < 0.001) (44). However,

in our study, there was also a significant difference at 2hr. The discrepancies may be due to the

difference in study population, study design, and other confounding variables.” In addition to

mentioned reasons, include type of surgery as main factor for discrepancy.

**Response:**Thank you for your observation and comments, we have corrected it and added what

the reviewer suggested.

**Comment 11:** I would recommend to paraphrase the limitation as follows:

This study was of high quality due to its double-blinded design; however, it has some limitations.

These include a lack of control over confounding variables such as incision size, the study being

monocentric, the timing of uterine exteriorization, and a shorter duration of postoperative follow-

up. Another limitation is that different obstetricians performed the wound infiltrations, which

may have resulted in variations in the procedure of wound infiltration technique..

**Response:** Thank you for your recommendations, we have corrected it as “This study was of

high quality due to its double-blinded design; however, it has some limitations. These include a

lack of control over confounding variables such as the size of the incision, being monocentric,

the time of uterine exteriorization, a shorter duration of postoperative follow-up, tramadol being

used for the management of shivering, previous cesarean section scar, the plasma levels of

medications not being determined, and different obstetricians performing wound infiltration.

We recommend that the researchers conduct further well-designed studies on parturients with

coexisting diseases and parturients undergoing emergency surgery.”

**Regards**

Mesay Milkias,

Semagn Mekonnen,

Hailemariam Getachew,

Hailemariam Mulugeta,

Siraj Ahmed,

Melkamu Kebede,

Belete Destaw,

Medhanit Melese,

Zemedu Aweke
