# Supplementary material for: Analgesic effectiveness of wound infiltration with bupivacaine versus a mixture of bupivacaine and tramadol for postoperative pain management among parturients undergoing elective cesarean section under spinal anesthesia: A randomized controlled trial
Source: PLoS One. 2025 Nov 12;20(11):e0336372. doi: 10.1371/journal.pone.0336372 (PMC12611141; doi:10.1371/journal.pone.0336372)
Supplement: Attachment — Submitted filename: Response Letter.docx [file pone.0336372.s007.docx]

**Response Letter**

**Dear Editor,**

We would like to thank the editor and reviewers for their careful and thorough reading of this manuscript and for their thoughtful comments and constructive suggestions, which helped us to improve the quality of this manuscript. We have read the research entitled “Analgesic Effectiveness of Wound Infiltration with Bupivacaine versus a Mixture of Bupivacaine and Tramadol for Postoperative Pain Management among Parturients Undergoing Elective Cesarean Section under Spinal Anesthesia”: A Double-Blind Randomized Controlled Trial

We have made all necessary corrections to the comments given by the editor and reviewers one by one as mentioned below. In addition, spelling, grammar, and syntax errors have been corrected.

**Editors comments**

**Comment 1:** Not all reviewer comments have been adequately addressed. I recommend that you carefully revisit them.

**Response:** Thank you, now we have addressed all the comments raised by the reviewers.

**Reviewer #1.** No comments.

**Reviewer #2**

**Comment 1:** Mesay Milkias Wonte and colleagues addressed some of the points, I raised but not others in my original review. Studies such as this one with vulnerable populations such as women who have given birth and have a responsibility to another person – their baby - have an ethical and medical responsibility to be as medically flawless as possible as other clinicians are liable to emulate their findings. This is one objective of studies. I am referring specifically to what seem to be controversies related to administering tramadol to women who have given birth and that the authors do not address this topic.

**Response:** Thank you for your insightful comments, we totally agree with your concerns, it was also our concern, however a lot of researches conducted in this group of patients shows that the site injection, route of injection, dose, frequency, and degree of metabolism determines the neonatal transfer of the drugs. The excretion of tramadol into milk is low and even lower amounts of the active metabolite, O-desmethyltramadol, are excreted. With usual maternal dosage, the amount excreted into breastmilk is much less than the dose that has been given to newborn infants for analgesia and is unlikely to adversely affect nursing infants(Palmer et al. 2018).

Seventy-five breastfed infants whose mothers were breastfeeding and taking tramadol 100 mg, every 6 hours following a cesarean section were compared to 75 matched infants at 2 to 4 days of age. Forty-nine percent of the mothers taking tramadol and all of the control mothers were taking other opiates (primarily oxycodone) and 61% of and 58%, respectively, also were taking a nonsteroidal anti-inflammatory agent (primarily diclofenac). Examination by a pediatrician revealed no difference between the groups using the Neurologic and Adaptive Capacity Score(K. F. Ilett et al, 2008). In our study tramadol was infiltrated at a wound site which has minimal rate of absorbtion in comparison to intravenous administration, but it significantly reduces pain scores, prolongs pain free state, and on top of that lowers 24-hour analgesia consumption. Here the tramadol was administered in diluted form and infiltrated subcuanseouly which will have minimal plasma concentration in comparison with other routes. Furthermore many studies are conducted in these area specifically on wound infiltration with a combination of opioids with local anesthetics but none of them reported any critical incidents in the mother as well as in the neonate.

**Comment 2:** Objective of the study: As I wrote in my original review, the authors still do not describe their study as a replication study and validation of infiltrating the C-Section incision with a mixture of tramadol and bupivacaine to their setting in Ethiopia. This seems to me a perfectly adequate aim. Though – as I wrote above - am not confident that administering tramadol in this population is medically justified.

**Response:** Thank you for your constructive comments, The primary objective of the study was to assesss the severity of pain at the 2^nd^, 6^th^, 12^th^, 18^th^, and 24^th^ hours. The secondary objectives were total analgesic consumption(mg), the time of the first analgesic request(minutes), incidence of shivering, and post-operative of nausea and vomiting.

- Replicating research studies offers several key benefits: validating results, ensuring quality, enhancing generalizability, and advancing scientific knowledge.
- Replication helps to confirm that findings are not due to chance or specific conditions and increases confidence in their reliability. It also allows for the identification of potential errors or biases in the original study.
- Similar studies were conducted in this population with the same interventions as we have used in developed countries; however no study was conducted in Ethiopia within this specific patient groups, so this randomized controlled trial was mainly aimed to address this issues. On the other hand it helped us to the check the validity and reliability of the previous study.
- Validation of this kind of study very important especially in resource limited-areas like our country Ethiopia to decrease the cost related to opioid purchasing frequency for postoperative pain which can have an economic impact on patients and hospitals.
- Eventhough tramadol is not recommended in pregnant mothers and lactating mothers, there are multitudes of studies which are conducted in this area including the systematic review but none of them reported any critical fetomaternal incidents associated with the tramadol infiltration at the wound site in normal healthy fit partuirents.

**Comment 3:** The authors still do not address the issue of which opioids are recommended vs not for women after C-Section and that there are warnings regarding use of tramadol. The authors assume that when the tramadol is infiltrated into the incision, absorption into the blood is probably not clinically significant. Is this assumption correct in pregnancy when vasculature is more extensive? However, even if this assumption is correct, after surgery, the tramadol was administered systemically. Whether orally or IV is not stated – this should be stated. I, therefore, suggest that the authors cannot avoid addressing the controversies related to tramadol.
Furthermore, it is possible that had they added an opioid to the spinal injection – rather than use a local anaesthesic only and administered other multi-modal treatments, eg 1-2 non-opioid groups round the clock - onset of pain may have been later and pain scores lower. I appreciate that this is a topic for a different study.

**Response:** Thank you for your insightful comments, we totally agree with you regarding the controversies. Generally tramadol is not recommended by FDA for pregnant mothers and lactating mothers, however the neonatal effects of this medication depends on several factors. Infant exposure to drugs in breast milk is dependent on the milk-to-plasma ratio, milk intake, and infant drug clearance (Fríguls B et al, 2010).

- By considering this we have selected appropriate parturients in inclusion criteria, term pregnant mothers to make sure that all fetal organs are fully matured, healthy fit mothers without any systemic illinesses to make sure that there is no problem in fetomaternal metabolism of drugs, and no history of opioid usage to make sure that the parturients plasma level of opioid is low or free. Daily infant dose can be calculated as the average concentration of drug in breast milk times the volume of milk ingested in 24 hours or by the milk-to-plasma ratio times maternal plasma concentration times the volume of milk ingested in 24 hours. In the breastfed infant, the estimated volume of milk is 150 mL/kg/day by day 3–4 of life (Salman S, et al. 2011).
- We have administered tramadol at wound site which have minimal absorption in comparison to other routes.
- The other thing we did to reduce the neonatal drug exposure was exclusion of parturients who were taking opioids in preoperative period to keep the maternal plasma level of opioid as low as possible.
- We have stated that the tramadol was given at incision site subcutaneously at a dose of 2mg/kg of tramadol in combination with bupivacaine (0.7mg/kg of 0.25%). None of the patients received tramadol orally or intravenously.
- We have searched for evidences regarding if there is any significant differences in subcutaneous changes between lactating mothers and other patient groups interms of vascularity, but no significant variation was observed. According to the studies majority of the changes are anatomical and physiological changes in major organs including, heart, lung, liver, GI, kidney, and brain. The dermatological changes are not significant.
- In our study we have not determined the plasma concentration of the drug but we have mentioned it in our limitation section section.
- Thank you for your recommendation we will consider addition of opioids to the spinal injections in our future researches.

**Comment 4:** Rather than calculate means - which can be misleading , particularly in studies where pain is assessed - the authors should add the proportion of women who reported scores of ≥3/10 at all the time points assessed. This is missing and the authors compared their findings of pain with the Kumar et al. who assessed the proportion of women who reported scores of >4 at rest and movement-related and did not find differences between the study groups.
The authors did not correct for age – the age range of the sample is large (15 to 49 years) and some underwent at least one C-Section. A child of 15 years old who has a C-Section will not respond to pain in the same way as a women who is 49 and this is her second or more C-Section. I appreciate that the groups are balanced, at least statistically, this respect.

**Response:** Thank you for your comments, we totally accept your comments, GEE is generally not ideal for making inferences about individual subjects, as it focuses on population-level effects and this is one of the limitation of GEE.

- To address this and avoid misinterpretation we have made the raw data fully accessible as supplementary file, so that the readers can easily access it and can effortlessly visualize and analyze the individual pain score at each time interval. We have analyzed the data with Generalized estimating equations(GEE) which has several advantages including flexibility in correlation structure, handling of missing data robustly, focus on population-Averaged effects, and computational effeciecy. That why we not provided individual pain scores, rather the average pain score at each time interval to exactly determine at which time the parturients feel pain. This average pain score at each time provides us a bias free interpretation of the results. Previously conducted studies analyzed their data with ANOVA which has a limitations when we compare it with the Generalized estimating equations.
- We have selected this age group (15-49) because the WHO categorized this age group as fertile age group. The parturients were allocated randomly to each group with computer generated codes and the data collectors were blinded to avoid selection bias. The socio-demographic characteristics of the patients including **age** was calculated but it was not statistically significant (P=0.45). That is why have not used correct for age.
- Regarding the number previous cesarean section, we totally agree with you and included it in the limitation section.

**Comment 5:** Minor Analgesic consumption – the units – mgs – is missing for average difference of tramadol between the groups. And for diclofenac. For tramadol - is a mean difference of 10 mg between the groups clinically significant ? The units of time are also missing under the mean difference for First Analgesia Request Time. Again – is a mean of 15 minutes difference between the groups, clinically significant? There is no need to write time at an accuracy of 3 digits after the decimal.

**Response:** Thank you for your detailed comment, we have corrected the units and milligrams.

- We have calculated effect size to determine the clinical significance(d=0.84), which is large effect size. To say clinically significant we have calculated the effect size(95% CI, 13.45 to 56.54), *t* (58) = 3.252, P= 0.002, **d=0.84**), not the mean difference. That is why we said it is clinically significant.
- Sorry, here we said it is statistically significant (95% CI, -182.087 to -119.913), *t* (58) = 5.6553, **P= 0.001**) not clinically significant. Because we have considered that p-value is statistically significant when the p-value less than 0.05.
- Thanks in advance we have corrected the decimals.

**Comment 6:** Under limitations: that authors should add that assessment of pain after surgery was not multi-modal, it included only assessing pain at rest. Minor - there is generally no need to repeat the actual findings in terms of data in the Discussion. This is the purpose of the Results section. Last but one paragraph on page 12 – the authors are probably referring to the systemic doses (IV? Oral?) of the tramadol and diclofenac. Again – no need to include the actual data –this was reported in the Results section.

**Response:** Thank you for your comments, we have added it in limitation section. Thanks in advance for your comments majority of journals including this journal recommends to describe the main findings of primary outcome variables in discussion section, that is why we have added some findings of primary outcome variables of research in the discussion section.

- In page 12, “This finding might be due to tramadol with bupivacaine acting synergistically, which might have provided lower analgesic consumption.” This statement was to describe that during wound infiltration with a mixture of tramadol and bupivacaine may act synergistically, that synergistic effect may prolong the analgesic duration, and believed to result in pain free period. It was not intravenous or oral route.
- The actual data was included, because according to the PLOS ONE submission guideline each and every underlying data have to be accessible freely for the public. That why we have included the actual data.

**Reviewer #3: No comments.**

**Regards**

Mesay Milkias,

Semagn Mekonnen,

Hailemariam Getachew,

Hailemariam Mulugeta,

Siraj Ahmed,

Melkamu Kebede,

Belete Destaw,

Medhanit Melese,

Zemedu Aweke
