# Supplementary material for: Analgesic effectiveness of wound infiltration with bupivacaine versus a mixture of bupivacaine and tramadol for postoperative pain management among parturients undergoing elective cesarean section under spinal anesthesia: A randomized controlled trial
Source: PLoS One. 2025 Nov 12;20(11):e0336372. doi: 10.1371/journal.pone.0336372 (PMC12611141; doi:10.1371/journal.pone.0336372)
Supplement: Attachment — Submitted filename: Response Letterrrr.docx [file pone.0336372.s008.docx]

**Response Letter**

**Dear Editor,**

We would like to thank the editor and reviewers for their careful and thorough reading of this manuscript and for their thoughtful comments and constructive suggestions, which helped us to improve the quality of this manuscript. We have read the research entitled “Analgesic Effectiveness of Wound Infiltration with Bupivacaine versus a Mixture of Bupivacaine and Tramadol for Postoperative Pain Management among Parturients Undergoing Elective Cesarean Section under Spinal Anesthesia”: A Double-Blind Randomized Controlled Trial

We have made all necessary corrections to the comments given by the editor and reviewers one by one as mentioned below. In addition, spelling, grammar, and syntax errors have been corrected.

**Editors comments**

**Comment 1:** The manuscript has improved during the second round of revisions. However, the second reviewers raised critical questions that the authors need to address.

**Response:** Thank you, now we have addressed all the comments raised by the second reviewer.

**Reviewer #1. No comments.**

**Reviewer #2**

**Comment 1:** The authors continue to avoid addressing the controversies related to tramadol in women who are pregnant and plan to breastfeed. The studies cited by the authors as indicating that tramadol is safe were not powered to detect side effects. The authors should be transparent about the use of tramadol in this cohort of women. Particularly if they wish that this form of treatment is to be employed by other clinicians.

**Response:** Thank you for your insightful comments, We appreciate the reviewer’s concern regarding tramadol use in lactating women. As noted, tramadol is not routinely recommended during breastfeeding due to potential neonatal risks. However, its use in our study was limited to a short duration in a setting where alternative opioids were unavailable. All mothers and neonates were closely monitored for difficulty in breast feeding, breathing difficulties, and drowsiness in the baby and no adverse effects were observed. We have added clarification in the manuscript to reflect this context and referenced relevant safety guidelines. This clinical trial may have significant contribution especially in resources limited set-ups, by minimizing the economic burden and side effects associated with higher dosages of opioids.

**Comment 2:** The authors admit that tramadol is not recommended in pregnant women and lactating mothers. The study has now been carried out and if the editors of this journal wish to publish it – it does not seem ethical to me to avoid addressing the complexities of administering tramadol in this cohort of women even when the medication is infiltrated to the surgical incision. Availability of other published studies – not powered to detect side effects – is not justification to continue along these lines. Furthermore, it is possible that women received tramadol systemically after the surgery. This is still unclear to me. Needs to be clarified.

**Response:** Thank you for your constructive comments, tramadol is not routinely recommended during breastfeeding due to potential neonatal risks, however this neonatal risks are minimized by minimizing the duration of drug exposure, reducing the dosage or administering lowest effective dose, and choosing the appropriate routes which have least absorption to breast milk are recommended. Almost all drugs are transferred into the breast milk to some extent. Infant exposure to drugs in breast milk is dependent on the milk-to-plasma ratio, milk intake, and infant drug clearance(Fríguls B., Joya X., García-Algar O., Pallás C.R., Vall O., Pichini S. (2010) A comprehensive review of assay methods to determine drugs in breast milk and the safety of breastfeeding when taking drugs. Analytical and Bioanalytical Chemistry, 397, 1157–1179).

- A trial in women who received a cesarean section randomized participants to receive 2 tablets of acetaminophen 325 mg and tramadol 35.5 mg (Zaldiar, Gruenthal, Israel) every 6 hours as well as diclofenac 100 mg at 12, 24 and 48 hours after arrival in the maternity ward, either as a fixed dose or the same drugs on demand. No differences were noted in neonatal outcomes of peak bilirubin, need for phototherapy, and adverse effects, including irritability or altered consciousness. No adverse infant effects were seen(Yefet E, Taha H, Salim R, et al. Fixed time interval compared with on-demand oral analgesia protocols for post-caesarean pain: A randomised controlled trial. BJOG 2017;124:1063–70.).
- A hospital in Japan provided postpartum mothers with a combination tablet containing tramadol 37.5 mg and acetaminophen 325 mg every 6 hours for 3 days if they requested a pain medication. A retrospective analysis found that of 148 mothers who received the drug, all infants were breastfed and none of the infants had an adverse reaction, such as drowsiness, difficulty breastfeeding or breathing problems(Nagae Seika P, Taizan K, Mariko S, et al. Safety of tramadol hydrochloride/acetaminophen combination tablet use for postpartum pain. J Obstet Gynaecol Res 2021;47:2919.). The relatively low dose and short duration of therapy may have reduced the risk of adverse reactions.
- Others clinical trial reported infants were exposed to <3% of a mother’s tramadol dose through breast milk with no evidence of harmful effects and suggested short-term maternal use was compatible with breastfeeding, This clinical study reported a milk-to-plasma ratio of 2.2 and 2.8 for tramadol and O-desmethyltramadol, respectively. Additionally, they reported a relative infant dose of 2.24 and 0.64% for tramadol and O-desmethyltramadol, respectively(Ilett K.F., Paech M.J., Page-Sharp M., Sy S.K., Kristensen J.H., Goy R., et al. (2008) Use of a sparse sampling study design to assess transfer of tramadol and its O-desmethyl metabolite into transitional breast milk. British Journal of Clinical Pharmacology, 65, 661–666.).

**Comment 3:** How was the tramadol (and diclofenac) after surgery administered? I cannot find that this is addressed in the Methods but it is in the Results, the authors write. How was this tramadol administered ?

**Response:** Thank you for your comment, In our study tramadol was used in the following scenarios:-

1. Following the surgery, the wound site was in the lower abdomen and transverse and about 1-2 inches above the pubic hairline, was infiltrated with 0.7 mg/kg of 0.25% bupivacaine diluted to 20 ml for group B. The BT group received a mixture of 0.7 mg/kg of 0.25% bupivacaine and 2 mg/kg of tramadol diluted to 20 milliliters by the four experienced obstetricians.
2. After the administration spinal anesthesia the grading of post-spinal shivering was assessed with Bedside Shivering Assessment Score (BSAS) and those patients whom develop shivering with BSAS grade greater or equal to 3 received intravenous route (IV) tramadol 0.5 mg/kg. The tramadol which adminstred for shivering may have little effect on our intervention, so we have mentioned it in the limitation section.
3. The mean tramadol consumption was lower in a mixture of tramadol and bupivacaine group (BT) (140.00+48.06 mg) than bupivacaine group (175.00+34.11 mg) with a statistically significant mean difference(MD) of 10.76 (95% CI, 13.45 to 56.54), t (58) = 3.25, P= 0.002, (d=0.84). Here tramadol was intravenously. There was no significant difference in mean diclofenac consumption between the two groups in a combination of tramadol and bupivacaine group (52.50+48.84 mg) than bupivacaine alone group (57.50+46.95 mg), (P=0.69), (d=0.10). Diclofenac was given intramuscular route(IM). Here we have mentioned the total analgesic given, because one of our secondary outcome was the total analgesia consumption.

**Comment 4:** The proportion of patients who report scores of 4/10 and above

I suggest that the authors create a table with the % of women in both groups whose scores are 4/10, displaying this information over time. This is a simple analysis to carry out and should not be expected of readers. To compare the pain scores, they might use Mann-Whitney U test and correct for multiple comparisons. The table can be included in the appendix as this is not the primary analysis.

**Response:** Thank you for your comments, we have added the table as supplementary file and corrected it; however we have used NRS to assess the severity of pain in our study which is categorical(Mild(1-3), Moderate(4-6), and Severe(7-10)), that is why have not provided the exact score as you mentioned above (4/10). In our study those parturients having pain score 4/10 is categorized under moderate.

**Comment 5:** In the Discussion, the authors cite the study carried out by Kumar M, Batra YK, Panda NB, Rajeev S, Nagi ON. Tramadol added to bupivacaine does not prolong analgesia of continuous psoas compartment block . Pain Pract. 2009;9(1):43-50. These were patients undergoing total hip replacement and received a different block - and so I am not sure how this citation is relevant for discussion of the findings in the current study, unless the authors intend to review different procedures where bupivacaine and tramadol are used for infusion. Please clarify.

**Response:** Thank you for your comment, we have removed it.

**Comment 6:** Replication study. The authors address the strengths of a replication study in their reply to the editor but do not address this in their manuscript. Women undergoing CSection in many countries in the world, would benefit from improved treatment and so it is laudable that the authors are seeking better care for women in Ethiopia.

**Response:** Thank you for your comments, we have added it.

**Reviewer #3: No comments.**

**Regards**

Mesay Milkias,

Semagn Mekonnen,

Hailemariam Getachew,

Hailemariam Mulugeta,

Siraj Ahmed,

Melkamu Kebede,

Belete Destaw,

Medhanit Melese,

Zemedu Aweke
