# Supplementary material for: Analgesic effectiveness of wound infiltration with bupivacaine versus a mixture of bupivacaine and tramadol for postoperative pain management among parturients undergoing elective cesarean section under spinal anesthesia: A randomized controlled trial
Source: PLoS One. 2025 Nov 12;20(11):e0336372. doi: 10.1371/journal.pone.0336372 (PMC12611141; doi:10.1371/journal.pone.0336372)
Supplement: Attachment — Submitted filename: Response_Letterrrr_auresp_4.docx [file pone.0336372.s009.docx]

**Response Letter**

**Dear Editor,**

We would like to thank the editor and reviewers for their careful and thorough reading of this manuscript and for their thoughtful comments and constructive suggestions, which helped us to improve the quality of this manuscript. We have read the research entitled “Analgesic Effectiveness of Wound Infiltration with Bupivacaine versus a Mixture of Bupivacaine and Tramadol for Postoperative Pain Management among Parturients Undergoing Elective Cesarean Section under Spinal Anesthesia”: A Double-Blind Randomized Controlled Trial

We have made all necessary corrections to the comments given by the editor and reviewers one by one as mentioned below. In addition, spelling, grammar, and syntax errors have been corrected.

**Editors comments**

**Comment 1:** The manuscript has notably improved following the third round of revisions, and the authors have addressed several previous concerns with greater clarity. However, Reviewer 2 has raised important and critical points that still require your attention prior to acceptance.

In particular, the safety of tramadol use in breastfeeding mothers remains insufficiently addressed. Please provide a clear, transparent, and evidence-based discussion on the potential risks associated with tramadol exposure in neonates through breast milk. This should include documented effects such as respiratory depression or central nervous system depression, supported by relevant and current literature. Ensure that this safety consideration is prominently discussed in both the manuscript and any clinical recommendations derived from your findings.

Additionally, please upload the supplementary table as referenced in your response letter or manuscript, as it is currently missing from the submission.

**Response:** Thank you, now we have addressed all the comments raised by the second reviewer regarding the safety of tramadol. Additionally we have added supplementary table which was missed during submission unintentionally, great apology for that.

**Reviewer #1. No comments.**

**Reviewer #2**

**Comment 1:** There is an ongoing to and fro with the authors about the safely of administering tramadol for lactating women.

As studies suggest that a significant proportion of people in Ethiopia may be CYP2D6 ultra-rapid metabolizers, medications such as tramadol and codeine increase the risk of adverse effects, especially in breastfeeding infants.

**Response:** Thank you for your insightful comments, We appreciate the reviewer’s concern regarding tramadol use in lactating women. As noted, tramadol is not routinely recommended during breastfeeding due to potential neonatal risks. However, its use in our study was limited to a short duration in a setting where alternative opioids were unavailable. All mothers and neonates were closely monitored for difficulty in breast feeding, breathing difficulties, and drowsiness in the baby and no adverse effects were observed. We have added clarification in the manuscript to reflect this context and referenced relevant safety guidelines as follows:-

- The safety of tramadol in neonates is limited due to the small size of the available data. Plasma concentrations of tramadol and its metabolite M1 in neonates, after breastfeeding, are influenced by three main factors: the amount of tramadol and M1 present in breast milk (which depends on maternal drug concentration, maternal metabolism, diffusion, ion trapping, and lipid partitioning), the quantity of breast milk consumed and its bioavailability, and the neonatal clearance of these compounds (Palmer GM, Anderson BJ, Linscott DK, Paech MJ, Allegaert K. Tramadol, breast feeding and safety in the newborn. Archives of disease in childhood. 2018;103(12):1110-3.).
- It is known that the amount of tramadol excreted into early breast milk is a small percentage of the maternal dose, specifically less than 2.5% per kg of body weight per day. In a study, seventy-five breastfeeding mothers were administered tramadol at a dose of 100 mg every 6 hours following a cesarean birth, in this study exposed infants and the control group of breastfed infants had similar characteristics, such as Apgar scores at birth and scores for neurological and adaptive capacities (Ilett KF, Paech, M. J., Page-Sharp, M., Sy, S. K., Kristensen, J. H., Goy, R., Chua, S., Christmas, T., & Scott, K. L. (2008). Use of a sparse sampling study design to assess transfer of tramadol and its O-desmethyl metabolite into transitional breast milk. British journal of clinical pharmacology,. 2008;65(5):661–6.).
- The neonate’s plasma concentration was 35 times higher than the upper limit found in breastfed infants of mothers prescribed 60 mg of codeine for postpartum pain (0.5–2.2 mcg/L) and 3.5 times the threshold that can cause respiratory depression in neonates (20 mcg/L). A review of the literature on tramadol use during breastfeeding has not identified any similar adverse events ((MD): DaLDLIB. National Institute of Child Health and Human Development; 2006- Tramadol [Updated 2025 Aug 15]. 2006.). While both tramadol and its M1 metabolite are present in breast milk, there is no evidence suggesting they carry the same risks associated with the ultra-rapid metabolism of codeine (Palmer GM, Anderson BJ, Linscott DK, Paech MJ, Allegaert K. Tramadol, breast feeding and safety in the newborn. Archives of disease in childhood. 2018;103(12):1110-3.).
- Several studies have been conducted regarding the safety of opioids in lactating mothers; however, they have small sample sizes, wide confidence intervals, and a high risk of bias ([https://www.nice.org.uk/guidance/ng192/evidence/f-opioids-for-pain-relief-after caesarean-birth-pdf-9071941651](https://www.nice.org.uk/guidance/ng192/evidence/f-opioids-for-pain-relief-after%20caesarean-birth-pdf-9071941651))
- In addition to this we have added DNA testing at limitation section to determine those patients which are ultrametabolizers in future studies.
- This clinical trial may have significant contribution to patients and family especially in resources limited set-ups like our country, by minimizing the economic burden and side effects associated with higher dosages of opioids.

**Comment 3:** How was the tramadol (and diclofenac) after surgery administered? I cannot find that this is addressed in the Methods but it is in the Results, the authors write. How was this tramadol administered. According to the authors:

1. It was infiltrated into the wound

2. For shivering – not clear was tramadol or pethidine administered for shivering ?

3. After surgery – IV tramadol with IM diclofenac. Why is this not stated clearly in the methods?

**Response:** Thank you for your comment, we have clearly stated how and when tramadol and diclofenac was used in our study. In our study tramadol was used in the following scenarios:-

1. Following the surgery, the wound site was in the lower abdomen and transverse and about 1-2 inches above the pubic hairline, was infiltrated with 0.7 mg/kg of 0.25% bupivacaine diluted to 20 ml for group B. The BT group received a mixture of 0.7 mg/kg of 0.25% bupivacaine and 2 mg/kg of tramadol diluted to 20 milliliters by the four experienced obstetricians. In this section tramadol was added to bupivacaine for the intervention group. (Page 6)
2. After the administration spinal anesthesia the grading of post-spinal shivering was assessed with Bedside Shivering Assessment Score (BSAS) and those patients whom develop shivering with BSAS grade greater or equal to 3 received intravenous route (IV) tramadol 0.5 mg/kg. The tramadol which adminstred for shivering may have little effect on our intervention, so we have mentioned it in the limitation section. Tramadol was used for shivering. In this section tramadol was given for shivering. (Page 6)
3. Thank you for observation, it was editorial problem, great apology for that, and we have corrected it. Standard care was provided equally for both groups postoperatively. Rescue analgesics were given based on the severity of pain, and the total analgesics(tramadol and diclofenac) administered during the first 24 hours are documented and analyzed as a secondary outcome.(Page 6)

**Reviewer #3: No comments.**

**Regards**

Mesay Milkias,

Semagn Mekonnen,

Hailemariam Getachew,

Hailemariam Mulugeta,

Siraj Ahmed,

Melkamu Kebede,

Belete Destaw,

Medhanit Melese,

Zemedu Aweke
